# Dilations of anastomotic strictures over time after repair of esophageal atresia

**DOI:** 10.1007/s00383-016-4013-z

**Published:** 2016-11-15

**Authors:** Pernilla Stenström, Magnus Anderberg, Anna Börjesson, Einar Arnbjörnsson

**Affiliations:** 1 Lund University, Lund, Sweden; 2 Department of Pediatric Surgery, Skåne University Hospital, Lund, Sweden

**Keywords:** Esophageal atresia (EA), Anastomotic stricture (AS), Balloon dilation, proton pump inhibitors (PPIs)

## Abstract

**Aim of the study:**

Anastomotic strictures commonly occur in patients undergoing surgery for esophageal atresia (EA). The primary aim of this study was to determine the age distribution of dilation procedures for anastomotic strictures over the patient’s childhood after reconstruction of EA. The secondary aim was to evaluate the effect of postoperative proton pump inhibitors (PPIs) on the frequency of dilations.

**Methods:**

This observational study was conducted at a single tertiary center of pediatric surgery. The times that dilations of strictures were performed were assessed during three study periods: 1983–1995, 2001–2009, and 2010–2014. PPIs were not used during the first period, and then, respectively, for 3 and 12 months postoperatively. The indications for dilation were signs of obstruction and/or radiological signs of stricture.

**Primary results:**

A total of 131 children underwent esophageal reconstruction, and of those, 60 (46%) required at least 1 dilation procedure for strictures. There were no differences in the frequencies of dilation procedures between the three study periods (28/66, 18/32 and 14/33, respectively; *P* = 0.42). The overall median number of dilations per patient was 3 (range 1–21) with no differences between the study periods. The differences between ages at which the first dilation was performed during each study period were significant, as follows: 7, 2, and 8 months, respectively (*P* = 0.03). Fiftyone percent of all dilation procedures were performed during the first year of life, 16% during the second year, and 33% during years 2–15. Four children (2%) underwent >12 dilations.

**Conclusion:**

The first year of life was the time of greatest need for dilation of AS after reconstruction of EA; however, dilations were also performed several years later. PPIs did not affect the frequency of dilations during the first year of life.

## Introduction

An anastomotic stricture (AS), which is a Clavian Dindo grade III complication [1], is reported to occur in 9–79% of newborns after reconstruction of esophageal atresia (EA) [2]. Proton pump inhibitors (PPIs) have been used to protect the esophageal anastomosis from acid gastroesophageal reflux in an attempt to diminish the risk of developing AS. However, evidence of the protective effect of PPIs is controversial [3, 4]. The timing of dilation procedures during the postoperative period, i.e., when and how often dilation is needed over the years, has not been reported. Information on the risk of AS, and the possible need of dilations for must be provided to the guardians of the affected neonates during initial counseling sessions. Therefore, detailed information on AS and its treatment is needed.

The primary aim of this study was to determine the number of times that dilation procedures were performed for AS in relation to the age of the child after reconstructive surgery for EA. The secondary aim was to evaluate the effect of postoperative proton pump inhibitors (PPIs) on the frequency of dilations during the first postoperative year.

## Materials and methods

Data were collected at a tertiary center of pediatric surgery, where reconstruction of EA has been performed since 1969. The center serves a region with a population that increased from 1.5 to 2.0 million residents over the study period.

All included children underwent primary anastomosis for type A or type C EA (without or with distal tracheoesophageal fistula). The surgeries occurred during three study periods (Table 1). For the periods 1983–1995 and 2001–2009, the information was collected retrospectively from charts. During 2010–2014, information was recorded in a prospectively collected database. The results from each EA treatment period were previously published [5–7].

**Table 1:** Summary of the results from three published studies performed at a single center. *EA* esophageal atresia, *AS* anastomotic stricture, *PPI* proton pump inhibitor

Study period	1983–1995 [5]	2001–2009 [6]	2010–2014 [7]	Sum	*P* value
Included children with EA, *n*	66	32	33	131	
Girls/Boys, *n*	28/38	8/24	9/24	45/86	0.16^a^
Treated with PPIs, *n*	0	32	33		
Length of PPI treatment (months)	0	3	12		
Number of children undergoing dilation due to AS, *n* (%)	28 (42)	18 (56)	14 (42)	60 (46)	0.42^a^
Stricture dilations, *n*	171	73	62	306	0.39^a^
Dilations per patient, median (range)	3 (1–21)	4 (1–20)	3 (1–15)	3 (1–21)	0.88^b^
Age at first dilation, months (range)	7 (1–39)	2 (1–12)	8 (1–11)	(1–39)	0.03^b^
Esophageal perforation during dilation procedure, *n* (%)	8 (12%)	0	1 (3%)	9	0.05^a^
Resection of anastomotic stricture, *n*	6	0	0	6	0.06^a^
Duration of follow up, years, median (range)	8 (2–16)	5 (1–10)	3 (1–6)		0.03^b^

Statistical methods

^a^Fisher exact test for a 2 × 3 contingency table

^b^Kruskal–Wallis test

This study collected data for the entire cohort of all three time periods. The main outcomes were the frequencies and times that dilations of AS were performed during the postoperative period of each patient up until each patient’s latest counseling session at the department.

AS was defined as a narrowing of the esophagus, identified on X-ray with contrast, and verified by esophagoscopy. Contrast esophagograms were routinely performed at 1–3, 6–8, and 12 months postoperatively, or following clinical suspicion of stricture formation (dysphagia, difficulty swallowing, and/or repeated vomiting).

Endoscopic dilation was performed with the patient under general anesthesia using CRE^®^ balloon dilators (Controlled Radial European Balloon Dilators; Boston Scientific, Watertown, MA, USA), and the GIFXP160^®^ video endoscope (Olympus). Dilation or calibration was performed no sooner than 3 weeks after the initial reconstruction and repeated at intervals of 2–3 weeks if needed, until the stricture had disappeared on esophagograms. During the first study period, only a resection of strictures followed by primary esophageal anastomosis was performed in six children.

Dilation was defined as a widening of the diameter of the AS. During balloon dilation, the balloon was inflated with contrast during fluoroscopic imaging. If the balloon contour was narrowed by the stricture, the procedure was considered to be dilation. If the balloon was not narrowed, the procedure was considered to be calibration. Calibrations were not included in the report.

### Statistical analysis

Statistical analysis was performed using the R software, version 3.2.0 (2015-04-16) (R Foundation for Statistical Computing). *P* < 0.05 was considered to be statistically significant.

### Ethical considerations

The study was performed according to the Declaration of Helsinki and approved by the Regional Ethical Review Board (registration number 2010/49). The data were coded and de-identified.

## Results

**Fig. 1:**
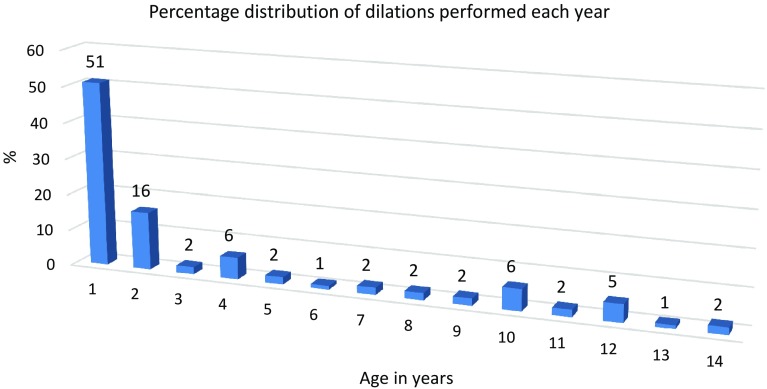
Number of dilations in percent performed at each year of age. The figure shows the percentages of dilations performed at each year of age of each pediatric study patient followed since birth. Dilations of anastomotic strictures after reconstruction for esophageal atresia occurred at all ages, but most frequently during the first 2 years of life (67%). The duration of time from reconstruction of the esophagus to the first dilation of the stricture was not significant between the three study periods (*P* = 0.37, Kruskal–Wallis test)

**Fig. 2:**
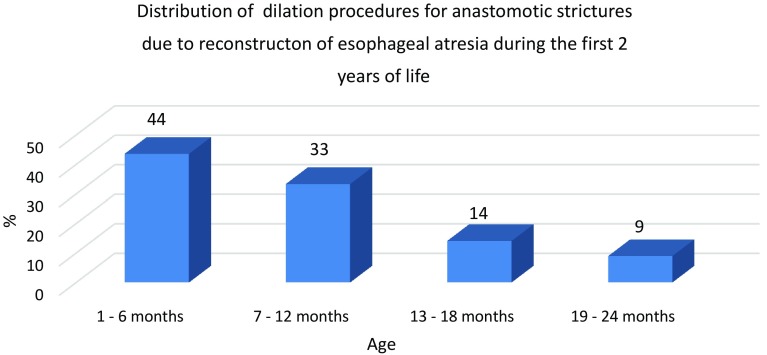
Ages of neonates at which dilation of an anastomotic stricture after reconstruction of esophageal atresia was performed. Percent dilation procedures, for 205 anastomotic strictures, during the first 2 years after corrective surgery for esophageal atresia performed during the first days of live

**Fig. 3:**
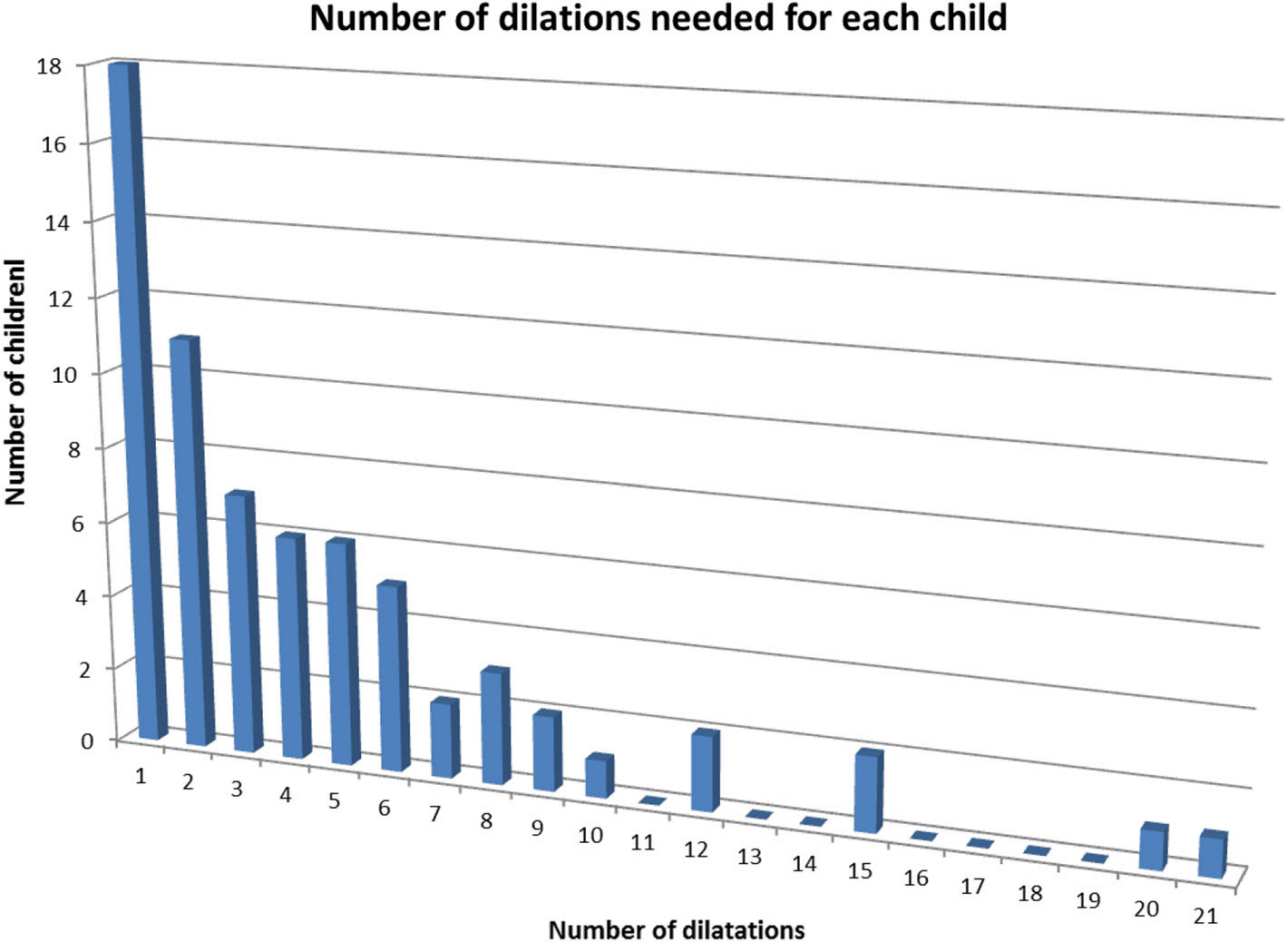
Number of dilations needed for each child. The figure summarizes the number of dilations needed for each child during the study periods. Only four children needed more than 12 dilations

A total of 131 children were included in the analyses of which 60 underwent dilatation. Dilation of AS occurred at all ages, ranging from 3 weeks to 15 years, but the majority, 205 (67%) of all 306 dilations, were performed during the first 2 years after reconstruction (Fig. 1). Of those, 44% out of 205 were performed within the first 6 months after the procedure (Fig. 2). The median number of dilations per study participant who underwent at least 1 dilation procedure was 3 (range 1–21). Four (2%) children required >12 procedures (Fig. 3).

The frequencies of dilations in the studied groups of children during the three different study periods ranged from 42 to 56%, without any significant differences. Perforation during dilation was more common during the first period. There were no differences in the median number of dilation procedures per patient between the three study periods, whereas differences in the median age at first dilation between the three periods were significant (Table 1). The duration of time from reconstruction of the esophagus of the newborn child to the first dilation of the stricture was significantly different between the three study periods (*P* = 0.37, Kruskal–Wallis test), Fig. 1 and Table 1.

## Discussion

The study found that dilation of AS was performed for 46% of 131 children with reconstructed EA. Dilation procedures were performed most frequently, 67%, of 306 dilations during the first 2 years after reconstruction; however, dilations were also needed later during childhood. Use of a PPI or duration of PPI treatment did not affect the frequency of dilations but the timing of the first dilation differed between the periods using various duration of PPI treatment, Table 1.

The rates of dilation procedures in our study are in the middle of a wide range of previously reported rates of dilations after esophageal atresia (9–79%) [2]. However, to the best of our knowledge, this is the first report on the timing of dilation procedures and for the duration of time after reconstruction that dilations might be necessary. Based on our results, dilations should be expected, especially for infants, but also for older children. Adequate information on the frequency of postoperative AS and the need for dilation is important, both with regard to parental counseling and also for planning the long-term postoperative care of patients with EA. Our results illustrate that parents to patients with EA must be informed about the risk of esophageal narrowing over time. Since some patients may also need dilations as adults, they should all be referred to specialists for adult care.

Previous reports [6, 7] showed that PPIs did not affect the frequency of dilations. Prospective randomized studies are needed to determine if the use of PPIs for decreasing the risk of AS is warranted. Results showing differences in the ages of children undergoing the first dilation between the study periods might be accounted for by progress in clinical and practical expertise over time instead of the effect of prophylactic PPIs. Alternatively, the management of signs of strictures might have changed to more conservative methods over time; either because longer treatment with PPIs resulted in more optimistic expectations, and/or that the inclusion in prospective database also influenced the clinical practice.

During the first study period, but not in the other study periods, resection of strictures followed by primary esophageal anastomosis was performed. Replacement of the affected esophagus with an interposition graft remains an option for AS refractory to all other forms of treatment, but was not used during the study periods. Adjuncts to dilation such as local steroid injection, topical application of mitomycin C, and esophageal stents were not used during the study periods and thereby do not confound the results. Currently, there is insufficient evidence to promote one adjunct therapy over another [2].

Since general anesthesia is needed for esophageal dilation procedures, and 5–10% of our study patients needed several procedures, the possibility of long-term sequelae of repeated anesthesia in young children should be considered [8, 9]. However, studies with long follow-ups that evaluated the cognitive dysfunction of patients undergoing repeated dilations of AS after EA reconstruction have not been reported and are urgently needed.

The strengths of this study were that all pediatric study patients were managed perioperatively at the same center and that outcomes were evaluated continuously. The limitations of this study are that the information was compiled both prospectively and retrospectively for the different time periods. Furthermore, the durations of follow-up periods varied between the three studies, and patients were not followed into adulthood.

Overall, the results presented here are of value for informing parents during counseling sessions and also for planning the pediatric surgical care of patients with EA, with regard to surgical volume and types of expected complications. Further studies are needed on the use of PPIs after EA repair, long-term effects of repeated anesthesia, and need for dilation procedures in adulthood.

## Conclusion

The frequency of dilation procedures for AS is highest during the first 2 years of life in patients who have undergone reconstruction for EA. PPI therapy does not affect the overall need for dilation.
